# Non-invasive intradialytic percutaneous perfusion monitoring: a view to the heart through the skin

**DOI:** 10.3389/fneph.2023.1124130

**Published:** 2023-07-11

**Authors:** Jarrin D. Penny, Lisa Hur, Fabio R. Salerno, Dickson Wong, M. Hussain Jan, Christopher W. McIntyre

**Affiliations:** ^1^ The Lilibeth Caberto Kidney Clinical Research Unit, London Health Sciences Centre, London, ON, Canada; ^2^ Department of Medical Biophysics, Western University, London, ON, Canada; ^3^ Lawson Health Research Institute, London, ON, Canada; ^4^ Division of Nephrology, London Health Sciences Centre, London, ON, Canada

**Keywords:** hemodialysis, cardiovascular injury, myocardial stunning, perfusion, ischemic injury

## Abstract

**Introduction:**

The life-sustaining treatment of hemodialysis (HD) induces recurrent and cumulative systemic circulatory stress resulting in cardiovascular injury. These recurrent insults compound preexisting cardiovascular sequalae leading to the development of myocardial injury and resulting in extremely high morbidity/mortality. This is largely a consequence of challenged microcirculatory flow within the myocardium (evidenced by detailed imaging-based studies). Currently, monitoring during HD is performed at the macrovascular level. Non-invasive monitoring of organ perfusion would allow the detection and therapeutic amelioration of this pathophysiological response to HD. Non-invasive percutaneous perfusion monitoring of the skin (using photoplethysmography—PPG) has been shown to be predictive of HD-induced myocardial stunning (a consequence of segmental ischemia). In this study, we extended these observations to include a dynamic assessment of skin perfusion during HD compared with directly measured myocardial perfusion during dialysis and cardiac contractile function.

**Methods:**

We evaluated the intradialytic microcirculatory response in 12 patients receiving conventional HD treatments using continuous percutaneous perfusion monitoring throughout HD. Cardiac echocardiography was performed prior to the initiation of HD, and again at peak-HD stress, to assess the development of regional wall motion abnormalities (RWMAs). Myocardial perfusion imaging was obtained at the same timepoints (pre-HD and peak-HD stress), utilizing intravenous administered contrast and a computerized tomography (CT)-based method. Intradialytic changes in pulse strength (derived from PPG) were compared with the development of HD-induced RWMAs (indicative of myocardial stunning) and changes in myocardial perfusion.

**Results:**

We found an association between the lowest pulse strength reduction (PPG) and the development of RWMAs (*p* = 0.03) and also with changes in global myocardial perfusion (CT) (*p* = 0.05). Ultrafiltration rate (mL/kg/hour) was a significant driver of HD-induced circulatory stress [(associated with the greatest pulse strength reduction (*p* = 0.01), a reduction in global myocardial perfusion (*p* = 0.001), and the development of RWMAs (*p* = 0.03)].

**Discussion:**

Percutaneous perfusion monitoring using PPG is a useful method of assessing intradialytic hemodynamic stability and HD-induced circulatory stress. The information generated at the microcirculatory level of the skin is reflective of direct measures of myocardial perfusion and the development of HD-induced myocardial stunning. This approach for the detection and management of HD-induced cardiac injury warrants additional evaluation.

## 1 Introduction

Chronic kidney disease (CKD) is often diagnosed when 50% of kidney function has been lost and a uremic environment is well established (1–5). Patients with CKD are at significant risk for cardiovascular (CV) morbidity/mortality—a risk that is 15 times greater than that in the general population (4, 6). Mortality rates are driven by pathophysiological processes shared by both the small and large vasculature (7, 8). A state of uremia, and associated co-morbid conditions (e.g., hypertension and diabetes) triggers a cascade of microcirculatory conditions which deteriorate vascular circulation (1, 9, 10). The continuous activation of the vascular endothelium creates an environment of chronic inflammation, thrombosis, and compromised vascular response (4), with levels of biomarkers being shown to progressively increase as kidney function declines (11). Consequently, structural and functional changes occur within the circulatory system, resulting in permanent damage and loss of compensatory mechanisms, and leaving patients vulnerable to hemodynamic instability (4, 12). At the microcirculatory level, poor tissue oxygenation and nutrient exchange occur within the tissues as capillary density and viability is lost—a precursor to the development of multiorgan vascular damage (i.e., to the skeletal muscle, kidneys, heart, brain, and gut) (1, 9).

**Figure 1:**
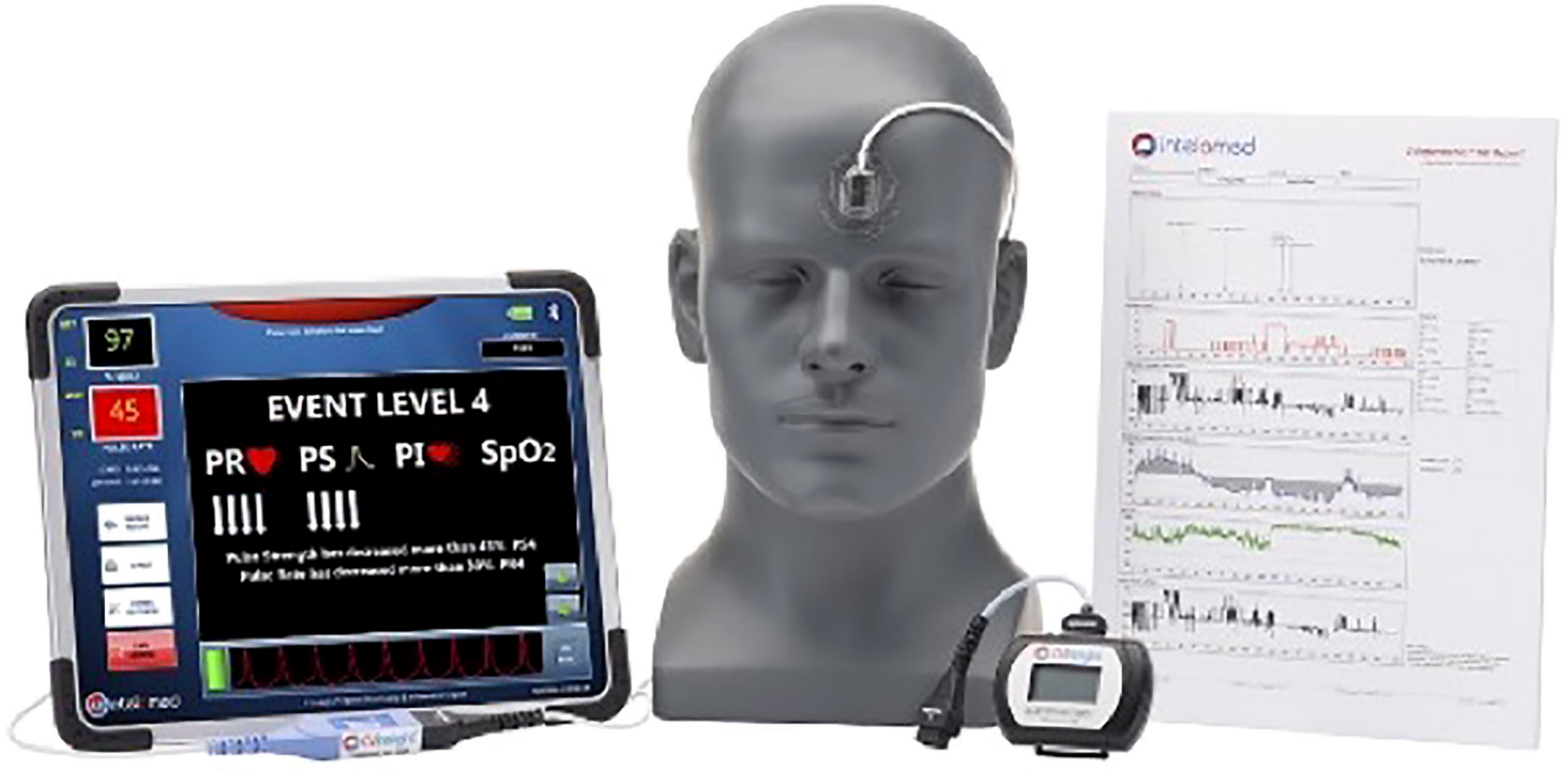
Picture of percutaneous perfusion monitoring system (CVInsight®).

The introduction of hemodialysis (HD) adds additional insult to this preexisting state, adding to extremes in morbidity/mortality (6, 13–15). HD patients are exposed to repetitive insults of demand myocardial ischemia/reperfusion injury as a result of HD/ultrafiltration and hypoperfusion (16). Recurrent episodes of ischemia precipitated by intermittent HD have negative consequences, leading to progressive myocardial damage and the development of non-viable myocardium and irreversible damage within months of starting HD. Many studies have described the phenomenon of HD-induced myocardial stunning—a common consequence of intermittent HD, directly associated with myocardial contractile dysfunction and patient survival (15–23). HD has been shown to be associated with reductions in global/segmental myocardial blood flow and the development of left ventricular regional wall motion abnormalities (RWMAs) (17, 17, 21, 22, 24, 25). The identification of myocardial stunning provides value for research purposes; however, due to serial echocardiography/*post hoc* analysis, clinical application is not viable. In a recent pilot study, however, our group evaluated the utility of a non-invasive percutaneous perfusion monitoring system for the detection of HD-induced myocardial stunning. The system continually assesses an individual’s unique CV status and response to HD using peripheral photoplethysmography (PPG). PPG uses infrared light, which is absorbed at the capillary level by oxy/deoxyhemoglobin proportionally to blood volume [CVInsight^®^ InteloMed Inc., Warrendale, PA—(**Figure 1**)]. The results were promising—PPG outputs were found to be predictive of HD-induced RWMAS/myocardial stunning—and no other associations between the development of myocardial stunning and conventional indices of intradialytic hemodynamic monitoring were found (26). HD treatments are currently driven by parameters specific to macrovasculature monitoring techniques, where subtleties of hemodynamic change either go without notice or are extremely latent responses.

The aim of our study was to further evaluate the utility of PPG as a predictor of HD-induced circulatory stress and the development of RWMAS. Furthermore, it was our intent to compare microcirculatory changes in skin perfusion (an accessible vascular bed) to direct measures of global myocardial perfusion changes during HD using intravenous contrast and computerized tomography (CT) imaging.

## 2 Materials and methods

This study was conducted according to GCP/ICH guidelines and the principles of the Declaration of Helsinki, with appropriate ethics committee approval. All patients gave their written, informed consent before participating in the study.

### 2.1 Study population

Twelve participants were recruited from the prevalent chronic HD population—London Health Sciences Center Renal Program, London, Ontario, Canada. Patients were included if they were > 18 years of age, receiving chronic HD therapy three times per week for > 3 months and had minimal to no urine output (< 250mL/24 hours).

### 2.2 Dialysis treatment details

Dialysis treatments were delivered in a single center (St. Joseph’s Hospital, London, Ontario, Canada) by a single operator (JDP). HD was administered using the Fresenius 5008 system, with high-flux polysulfone dialyzer and according to the participant’s routine HD prescription. Treatments were delivered midweek (Wednesday or Thursday) during the short interdialytic period. The majority of the treatments lasted for 4 hours (10/12); however, two patients received slightly shorter treatments. Dialysate parameters were programmed according to patient’s individual prescriptions; the dialysate sodium range was between 137 and 140 mmol/L; the dialysate potassium was either 1.5 or 3 mmol/L; all patients had a dialysate calcium of 1.25 mmol/L; and bicarbonate ranged from 35 to 40 mmol/L. Anticoagulation was achieved using low-molecular-weight heparin (Dalteparin), with doses between 2,500 and 5,000 units. Dialysate flow was 500mL/minute, and the temperature was set to 36.5°C for all treatments. For each session, net ultrafiltration was set on an individual basis according to the patients’ achievable ideal dry weight. Blood pump speed varied between 330 and 400mL/minute. Four participants were dialyzed via arteriovenous fistula, one via loop graft, and the remaining seven via a central venous catheter. Intradialytic weight gain ranged from 1.1–2.8 kg (see **Table 1** for details). Dialysis monitoring parameters were obtained in accordance with program policy with intradialytic measures documented every 30 minutes and more often as required. Intradialytic hypotension (IDH) was defined as a reduction in systolic blood pressure (SBP) of ≥ 20 mmHg and/or ≤ 100 mmHg in association with typical symptoms of hypotension, such as nausea, lightheadedness, or cramping, requiring intervention by a care provider.

**Table 1:** Patient demographics and dialysis treatment details.

Age (years)	67.2 ± 13.4
Hemodialysis vintage (months) 3–6 RWMAS 7–12 RWMAS	54.8 ± 40.7 48 ± 39 77.6 ± 17.7
Gender (M/F)	9/3
Diabetes (Y/N)	6/6
History CHF (Y/N)	3/9
History CAD (Y/N)	6/3
ACE/ARB (Y/N)	4/8
Beta blocker (Y/N)	7/5
Statin (Y/N)	9/3
Hemoglobin (g/L)	98.8 ± 9.9
Urea (mmol/L)	18.3 ± 5.9
Creatinine (mmol/L)	765.7 ± 209.9
Sodium (mmol/L)	134.2 ± 2.8
Potassium (mmol/L)	4.4 ± 0.7
Albumin (g/L)	37.8 ± 2.5
Treatment time (minutes)	232.5 ± 16.4
Pre-HD systolic BP (mmHg)	143.3 ± 22.3
Pre-HD diastolic BP (mmHg)	64 ± 13.7
Nadir systolic BP (mmHg)	101.3 ± 15.9
IDWG (kg)	1.8 ± 0.66
UF Rate (mL/hour)	735.6 ± 174.8
UF (mL/kg/hour)	8.5 ± 2.9
Minimum RBV (%)	84.7 ± 4.6
Intradialytic symptoms (Y/N)	1/11
Intradialytic interventions (Y/N)	8/4
IDH (Y/N)	0/0
Intradialytic systolic BP reduction (mmHg)	41.7 ± 14.5
Age (years)	67.2 ± 13.4
Hemodialysis vintage (months) 3–6 RWMAS 7–12 RWMAS	54.8 ± 40.7 48 ± 39 77.6 ± 17.7
Gender (M/F)	9/3
Diabetes (Y/N)	6/6
History CHF (Y/N)	3/9
History CAD (Y/N)	6/3
ACE/ARB (Y/N)	4/8
Beta blocker (Y/N)	7/5
Statin (Y/N)	9/3
Hemoglobin (g/L)	98.8 ± 9.9
Urea (mmol/L)	18.3 ± 5.9
Creatinine (mmol/L)	765.7 ± 209.9
Sodium (mmol/L)	134.2 ± 2.8
Potassium (mmol/L)	4.4 ± 0.7
Albumin (g/L)	37.8 ± 2.5
Treatment time (minutes)	232.5 ± 16.4
Pre-HD systolic BP (mmHg)	143.3 ± 22.3
Pre-HD diastolic BP (mmHg)	64 ± 13.7
Nadir systolic BP (mmHg)	101.3 ± 15.9
IDWG (kg)	1.8 ± 0.66
UF Rate (mL/hour)	735.6 ± 174.8
UF (mL/kg/hour)	8.5 ± 2.9
Minimum RBV (%)	84.7 ± 4.6
Intradialytic symptoms (Y/N)	1/11
Intradialytic interventions (Y/N)	8/4
IDH (Y/N)	0/0
Intradialytic systolic BP reduction (mmHg)	41.7 ± 14.5

RWMAs, regional wall motion abnormalities; M, male; F, female; Y, yes; N, no; CHF, congestive heart failure; CAD, coronary artery disease; ACE, ace inhibitor; ARB, angiotensin receptor blocker; HD, hemodialysis; BP, blood pressure; IDWG, interdialytic weight gain; UF, ultrafiltration; RBV, relative blood volume; IDH, intradialytic weight gain.

### 2.3 Continuous cutaneous perfusion monitoring

The forehead area of interest was cleansed using 70% isopropyl alcohol. The optical oximetry sensor was placed on the patient’s forehead midline, approximately 2.5 cm above the level of the nose. Once securely attached, the patient was asked to sit comfortably in their typical dialysis position (e.g., feet elevated, chair reclined) for approximately 5 minutes to establish a resting state, at which point a baseline PPG measurement was captured. Baseline measures are taken (a) to ensure proper sensor placement and adequate PPG signal and (b) as the basis for individualized hemodynamic comparison of variations throughout HD. Continuous hemodynamic data were then captured throughout the entire HD treatment. Any HD events/interventions were annotated on the CVI monitoring device for each participant, including the initiation and completion of HD, timing of echocardiogram and CT, and symptoms experienced by the participant (e.g., dizziness, lightheadedness, cramping, nausea, headache, pain/discomfort, decrease in blood pressure, or any interventions delivered by the care provider such as ultrafiltration changes, position change or fluid resuscitation requirements). CVI-derived variables were extracted from the CVI-generated output files on a case-by-case basis using a customized semi-automated pipeline designed using R Studio (R Core Team (2022). R: A language and environment for statistical computing. R Foundation for Statistical Computing, Vienna, Austria. URL: http://www.R-project.org/ and RStudio Team (2022). RStudio: Integrated Development for R. RStudio, PBC, Boston, MA URL http://www.rstudio.com/) using the *tidyverse* package (27). All acquired data were analyzed *post hoc* (FRS, MHJ, DW JDP).

### 2.4 CT perfusion—dynamic contrast enhanced CT acquisition

Dynamic CT images of the heart were acquired at baseline and peak HD treatment timepoints. Participants were aligned on a CT bed in supine position and scans were performed between 75%–75% R–R interval, prospectively ECG-gated while the participants were free breathing. For the quantification of myocardial blood flow, iodinated contrast agent (Isovue 370) was delivered intravenously during the image acquisition and the delivery of contrast was traced with 32 scans every 1–2 heartbeats. The scanner setting for all dynamic CT images are as listed: display field of view = 45.0 cm; tube voltage = 100–120 kV; tube current = 100 mA; detector coverage = 160 mm; gantry period = 0.28 s; slice thickness = 2.5 mm.

Following the imaging visit, the dynamic images were processed (LH) utilizing the proprietary ASiR algorithm (Resolution CT console, GE Healthcare) to alter the slice thickness to 2.5 mm with the aim of increasing the signal-to-noise ratio. The reconstructed images were correlated for residual cardiac and respiratory motion using a 3D non-rigid registration algorithm on a proprietary workstation (GE proprietary software, advantage workstation, GE Healthcare). Myocardial blood flow maps of the dynamic images were generated with the application of the Johnson–Wilson–Lee model of tracer kinetics for each HD timepoint (28). Seven slices of the left ventricular myocardium were selected and delineated for the absolute measurement of global myocardial blood flow. For each timepoint, the seven slices were averaged, and the mean global myocardial blood flow measurements were recorded for analysis.

### 2.5 Echocardiography

Echocardiography was performed by a trained investigator (LH) prior to commencing HD and again at peak-HD stress (i.e., approximately 20 minutes prior to the end of HD) using commercially available equipment (1.5–3.6 MHz M4S probe, Vivid-q, GE Medical Systems, Soningen, Germany). Standard apical two- and four-chamber views of the left ventricle were recorded for offline analysis using semi-automated software (EchoPac, GE Healthcare)—2D speckle tracking software. Images were anonymized and analyzed in a random order by a trained investigator (JDP), and a random sample of these images was analyzed in random order by a second appropriately trained investigator (LH) to determine estimates of interobserver reliability. Three cardiac cycles were analyzed for each timepoint and the segmental strain values were derived from 12 left ventricular segments. Myocardial segments with a ≥ 20% reduction in longitudinal strain (between pre- and peak-HD stress) were determined to have developed a HD-induced regional wall motion abnormality (RWMA). The presence of two or more RWMAs was defined as myocardial stunning in accordance with previously published methods (17, 22). Poor-quality images were removed from the analysis, and any segment that was not visible or in which the software was not able to accurately track speckles was not counted as an RWMA.

### 2.6 Statistical analysis

Statistical analysis was performed using JASP [Netherlands (version 0.14.1)]. Descriptive statistics are expressed as mean standard deviation, median or percent. All data were tested for normality using the Shapiro–Wilk test. Comparisons of related outcomes at two different timepoints were performed using the paired t-test for parametric data and the Wilcoxon signed-rank test for non-parametric data. Bivariate correlation was assessed using Pearson’s correlation coefficient for parametric data and Spearman’s coefficient for non-parametric data. An alpha error of less than 5% (*p* < 0.05) was statistically significant. Graphs were created using Prism GraphPad (version 9.4.0).

## 3 Results

### 3.1 Baseline characteristics

Baseline clinical characteristics are shown in **Table 1**. All participants received conventional chronic HD treatment three times per week. All participants were anuric. The mean age of our population was 67.2 ± 13.4 years, the mean dialysis vintage of the population was 54.8 ± 40.7 months, and nine participants were male. The causes of renal failure included hypertension (58%) and diabetes (33%). Other causes included IgA nephropathy, hepatorenal syndrome, and toxicity. Other comorbidities included coronary artery disease (50%) and congestive heart failure (25%). All patients were taking either monotherapy or combination antihypertensive/cardiac medication (**Table 1**). The mean systolic BP prior to the initiation of HD was 143.3 ± 22.3 mmHg, the diastolic BP was 64 ± 13 mmHg, and the intradialytic nadir systolic BP was 101.3 ± 15.9 mmHg. The average interdialytic weight gain was 1.8 ± 0.66 kg, the mean ultrafiltration (UF) rate was 735.6 ± 174.8 mL/hour, and the mean UF mL/kg/hour was 8.5 ± 2.9. One participant had intradialytic symptoms due to vasal vagal response to choking/coughing (preexisting issue). This was not related to intradialytic hypotension; however, a 200 mL bolus of fluid was delivered due to brief unresponsiveness. Seven treatments required reductions to UF rate related to non-symptomatic reductions in BP. There were no treatments that met our definition of IDH. The average intradialytic reduction in systolic BP was 41.7 ± 14.5 mmHg in the absence of symptoms, with a mean relative blood volume (RBV) reduction of 41.7% ± 14.5%.

### 3.2 Percutaneous perfusion monitoring

The PPG waveform produced an embedded raw pulse strength (PS) measurement at baseline. From this baseline measurement, the mean intradialytic PS change was −10.9% ± 33.8%, whereas the lowest PS reduction from baseline was found to be −57.5% ± 22.2%. On average, it took 119.7 ± 53.5 minutes for participants in the population to reach their lowest PS threshold. Participants spent on average 61.6% ± 34.4% of HD treatment with a PS reduction of 10% from baseline. Ten participants had a further reduction in PS of 20% from baseline, spending 53.5% ± 32% of HD treatment at that threshold. The same participants had a further PS reduction of 30% and 40% for 40.6% ± 29.6% and 28.3% ± 24.6% of HD, respectively. A further PS reduction of 50% was observed in nine participants, who spent 15.2% ± 18.2% of HD at this threshold (**Table 2**).

**Table 2:** Descriptive statistics for measures hemodialysis-induced circulatory stress.

	Mean ± SD	Median	Range
Change in PS (%)	−10.9 ± 33.8	−24.7	-46.5 to 62.9
Lowest PS reduction (%) 3–6 RWMAs 7–12 RWMAs	−57.5 ± 22.2 −47 ± 25.9 −67.9 ± 12.5	−52.7 −60.5 −70.2	-10 to -83 -10.5 to -70 -46.4 to -83
Time to lowest PS (minutes)	119.7 ± 53.7	130.2	5.5 to 100.6
Time spent with PS below −10 (%) 3–6 RWMAs (%) 7–12 RWMAs (%)	61.6 ± 34.4 48.4 ± 38.5 78.8 ± 26.6	73.5 62.8 85.7	0.1 to 98.4 0.1 to 87.1 37.2 to 98.4
Time spent with PS below −20 (%) 3–6 RWMAs (%) 7–12 RWMAs (%)	53.5 ± 32 40 ± 32.8 65.1 ± 28.8	60.1 48.9 75.7	0 to 91.3 0 to 76 29.6 to 91.3
Time spent with PS below −30 (%) 3–6 RWMAs (%) 7–12 RWMAs (%)	40.6 ± 29.6 30 ± 26.3 51.3 ± 31.2	42.9 32.2 59	0 to 81.7 0 to 64.5 6.5 to 81.7
Time spent with PS below −40 (%) 3–6 RWMAs (%) 7–12 RWMAs (%)	28.3 ± 24.6 19.1 ± 19.1 37.5 ± 27.7	23.1 16.1 37.8	0 to 3.5 0 to 48.2 0.5 to 75.5
Time spent with PS below −50 (%) 3–6 RWMAs (%) 7–12 RWMAs (%)	12.2 ± 18.2 6.5 ± 7.5 23.9 ± 22.2	73.9 3.6 22.1	0 to 57.2 0 to 17.5 0 to 57.2
Number of RWMAs	6 ± 2.9	6.5	3 to 12
Change in GP (%) 3–6 RWMAs (%) 7–12 RWMAs (%)	−17.1 ± 16.9 −12 ± 15.2 −21.5 ± 17.7	−15.1 −14 −16	-52.4 to 0.2 -23.5 to 3.6 -6.8 to -52.4
GP pre HD (mL/minute/100 g)	93.9 ± 28.5	83.8	59.8 to 41.2
GP peak-HD stress (mL/minute/100 g)	74.2 ± 13.3	70.9	57.8 to 101.8

PS, pulse strength; HD, hemodialysis; RWMAs, regional wall motion abnormalities; GP, global perfusion.

### 3.3 HD-induced myocardial ischemic injury and relationship to percutaneous perfusion

**Figure 2:**
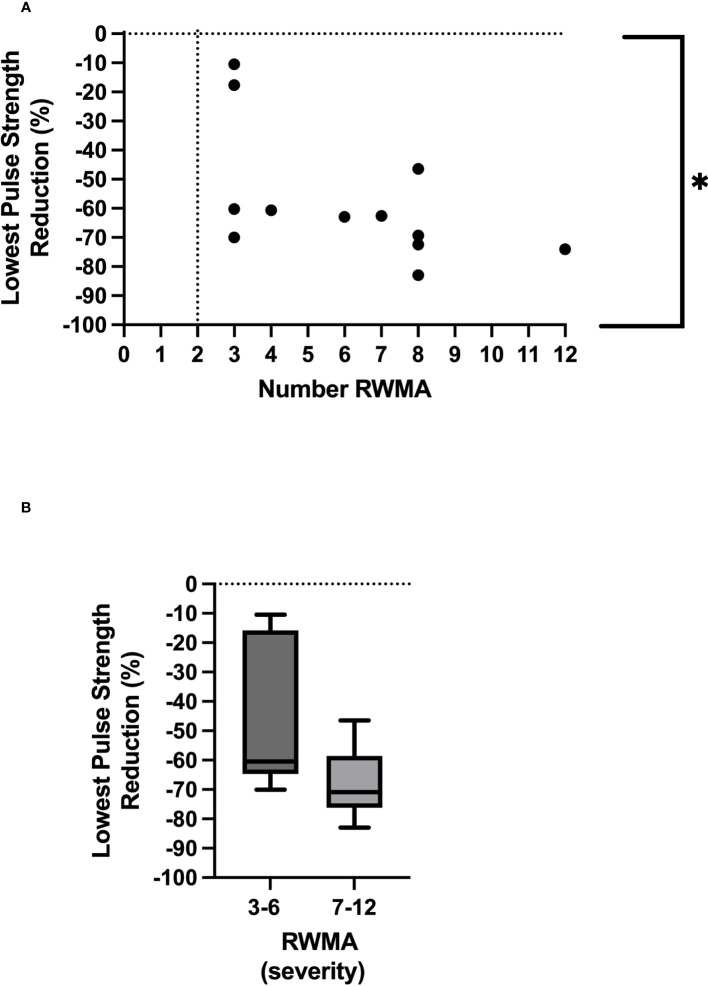
Relationship between lowest pulse strength reduction and number of regional wall motion abnormalities *Denotes *p* = 0.03 **(A)**. Severity of RWMAS and lowest pulse strength reduction (*p* = 0.09) **(B)**.

All 12 participants exhibited treatment-induced myocardial ischemic injury, defined as myocardial stunning. The number of left ventricular segments that underwent a 20% reduction in longitudinal strain ranged between three and 12, mean 6.1 ± 2.9 (**Table 2**). The lowest PS reduction during HD was associated with the development of RWMAs [*p* = 0.03, r = 0.63 (**Figure 2**)]. In addition, there were trending patterns in terms of time spent at PS reduction thresholds and the number of RWMAs, with statistical significance being reached with a PS reduction of 50% from baseline [(*p* = 0.048, r = 0.58), **Tables 2**, **3**]. When stratifying participants by the mean number of RWMAs (severity), although no statistical significance was reached between groups, those who developed a higher number of RWMAs had a lower PS reduction [3–6 RWMAs, PS −47.4 ± 25.9; 7–12 RWMAs, PS −67.9 ± 12.5, *p* = 0.09 (**Table 2**; **Figure 2B**)] and spent more time at each PS threshold (**Table 2**). In addition, there was a greater reduction in global myocardial perfusion in those with an increased number of RWMAs. Furthermore, increasing numbers of RWMAs were associated with longer HD vintage [7–12 RWMAs = 77.6 ± 17.7 months, whereas 3–6 RWMAs = 48 ± 39 months (**Table 1**)].

**Table 3:** Correlations between measures of global perfusion, measures of HD-induced circulatory stress and HD demographics/parameters A. Between group comparison B.

			*p*-value	Correlation
A	Percent change GP	Lowest PS reduction	0.048*	0.58
	Percent change GP	Pre-HD systolic BP	0.144	0.45
	Percent change GP	Systolic BP reduction	0.451	0.24
	Percent change GP	Nadir BP	0.151	0.44
	Percent change GP	Minimum RBV	0.49*	0.58
	% treatment PS −20%	Number of RWMAs	0.055	0.59
	% treatment PS −30%	Number of RWMAs	0.058	0.56
	% treatment PS −40%	Number of RWMAs	0.052	0.57
	% treatment PS −50%	Number of RWMAs	0.048*	0.58
	UF mL/kg/hour	Percent change GP	0.001***	−0.83
	UF mL/hg/hour	Number of RWMAs	0.026*	0.64
	UF mL/kg/hour	Time spent PS −10%	0.032*	0.063
	UF mL/kg/hour	Time spent PS −20%	0.033*	0.61
	UF mL/kg/hour	Time spent PS −30%	0.066	0.55
	UF mL/kg/hour	Time spent PS −40%	0.144	0.45
	UF mL/kg/hour	Lowest PS reduction	0.003**	−0.78
	Mean UFR	Percent change GP	0.028*	−0.64
	Lowest PS reduction	Number of RWMAs	0.030*	−0.63
	Lowest PS reduction	Pre-HD systolic BP	0.560	0.19
	Lowest PS reduction	Nadir BP	0.974	0.01
	Lowest PS reduction	Systolic BP reduction	0.564	0.19
	Lowest PS reduction	Minimum RBV	0.534	0.20
B	GP pre HD	GP peak HD stress	0.002**	

UF, ultrafiltration; mL, milliliters; kg, kilogram; RWMAs, regional wall motion abnormalities; RBV, relative blood volume; PS, pulse strength; BP, blood pressure; GP, global perfusion; HD, hemodialysis. *P ≤ 0.05, **P ≤ 0.01, ***P ≤ 0.001.

### 3.4 Direct measures of cardiac perfusion and relationship to percutaneous perfusion

**Figure 3:**
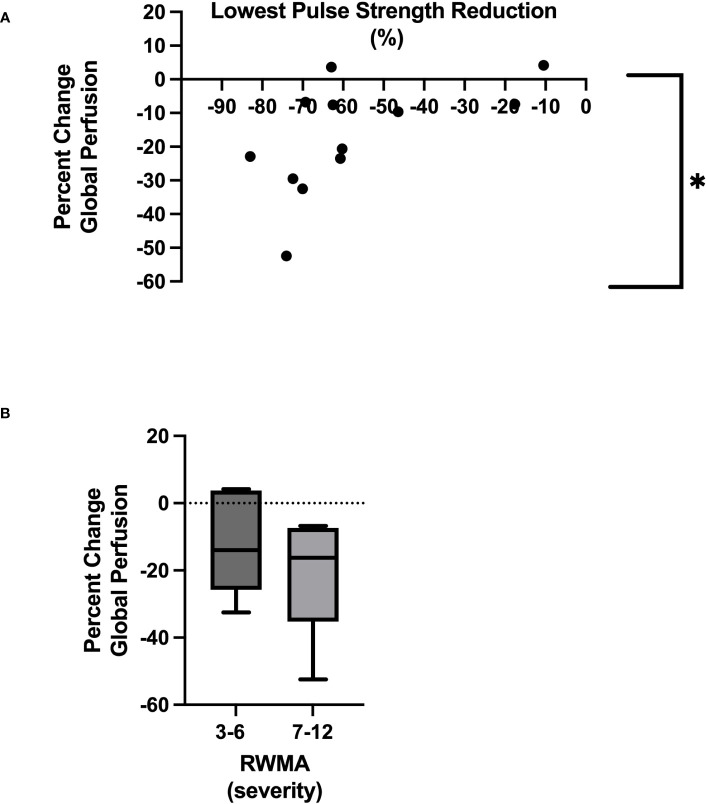
Relationship between lowest pulse strength reduction and global myocardial perfusion *Denotes *p* = 0.05 **(A)**. Severity of RWMAS and reduction in global myocardial perfusion (*p* = 0.48) **(B)**.

The mean global perfusion before HD was 93.9 ± 28.5 mL/minute/100 g, whereas the mean global perfusion at peak-HD stress was reduced to 74.2 ± 13.3 mL/minute/100 g [(*p* **=** 0.002), **Tables 2**, **3**]. The average change in global perfusion between the two timepoints was a reduction of −17.1% ± 16.9% (**Table 2**). This trend was also observed in 10 participants, in whom we observed a reduction in global cardiac perfusion at peak-HD stress; however, in two participants, we observed a very slight increase in global perfusion (3.6mL/minute/100 g, 4.2mL/minute/100 g). Direct changes in intradialytic global myocardial perfusion were associated with reductions in PS [*p* **=** 0.048, r **=** 0.58 (**Table 3**; **Figure 3A**)]. In addition, patients who developed more RWMAs also had a lower reduction in global perfusion [3–6 RWMAs, global perfusion −12 ± 15.2; 7–12 RWMAs, global perfusion −21.5 ± 17.7, *p* **=** 0.48 (**Table 2**; **Figure 3B**)].

### 3.5 Relationship to ultrafiltration

**Figure 4:**
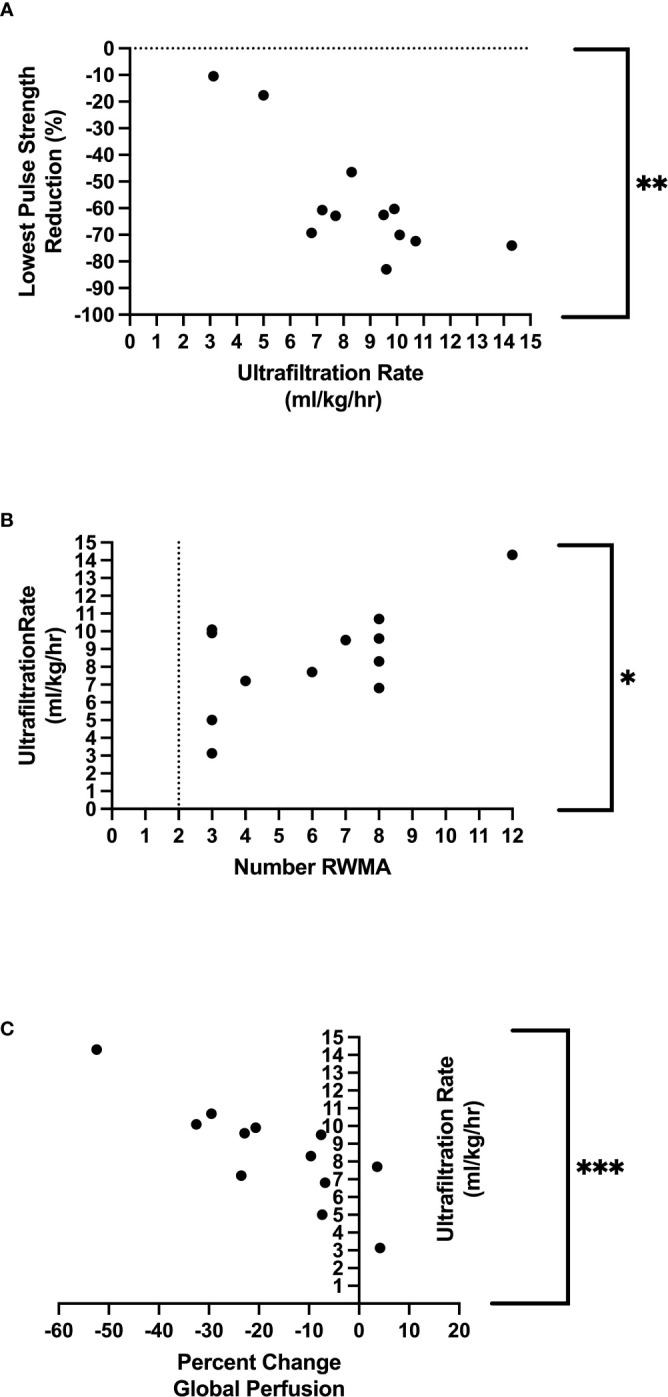
Relationship between ultrafiltration and lowest pulse strength reduction **Denotes p=0.003 **(A)**, relationship between ultrafiltration and regional wall motion abnormalities *Denotes *p* = 0.03 **(B)**, relationship between ultrafiltration and global perfusion ***Denotes *p*=<0.001 **(C)**.

UF rates were set in a linear profile using the patients’ achievable prescribed ideal body weight. The mean UF rate for the group studied was 735.6 ± 174.8 mL/hour, equating to 8.5 ± 2.9mL/kg of body weight per hour (mL/kg/hour), which was used for the following correlations seen in **Table 3**. Rate of fluid removal was associated with percutaneous perfusion measures of lowest PS reduction [*p* **=** 0.003, r **=** −0.78 (**Figure 4A**)], time spent at 10% reduction threshold (*p* **=** 0.033, r **=** 0.63), and time spent at 20% reduction threshold [(*p* **=** 0.034, r **=** 0.61), **Table 3**]. UF was also associated with the number of HD-induced RWMAS [*p* **=** 0.026, r **=** 0.64 (**Figure 4B**)], and also with direct changes in myocardial perfusion [*p* **=** 0.001, r **=** −0.83 (**Figure 4C**)]. Notably, increased myocardial perfusion was related to a very minimal ultrafiltration requirement in one participant.

### 3.6 Standard HD parameters

There were no relationships found among population demographics and lowest PS reduction nor global perfusion—including age (*p* = 0.174 and *p* = 0.313, respectively) and HD vintage (*p* = 1.0 and *p* = 0.543, respectively). There were also no associations between traditional macrovascular measures of HD-associated stability and reductions in PS—including pre-HD systolic BP (*p* = 0.560), intradialytic systolic BP reduction (*p* = 0.564), nadir BP (*p* = 0.974), or minimum RBV (*p* = 0.534). In addition, we found no relationship among pre-HD systolic BP, intradialytic systolic BP reduction, nadir BP, and changes in global perfusion (*p* = 0.144, *p* = 0.451, and *p* = 0.151, respectively). There was, however, a slight association between changes in global perfusion and relative blood volume [RBV (*p* = 0.049, r = 0.58)]. Details can be found in **Table 3**.

## 4 Discussion

This study further confirms that percutaneous perfusion monitoring (using PPG) is a useful method for assessing intradialytic hemodynamic stability and HD-induced circulatory stress. In addition, the information generated at the microcirculatory level of the skin is reflective of direct measures of myocardial perfusion and the development of HD-induced myocardial stunning.

Current results confirm previous findings (using the same technology) that identified the variable PS as a key PPG parameter that signals the development of HD-induced circulatory stress and myocardial stunning at the microcirculatory level (26). The PS parameter represents the delivered pulsatile blood volume to the capillaries of the skin with each heartbeat and is reflective of stroke volume and microcirculatory tissue perfusion. In the critical care setting, accessible vascular beds (sublingual mucosa) were used as surrogates for vital organ perfusion (gut) (29–31), indicating that microcirculatory resuscitation was directly associated with clinical outcomes—whereby survivors had a restoration of microcirculatory perfusion, and non-survivors did not (independent to large vessel indices) (30, 32–36). During HD, extracorporeal redistribution, ultrafiltration, and reduced circulatory volume reflect a negative PPG waveform where PS reduction from baseline (before the initiation of HD) progresses over the course of HD as volume is removed (alternatively, a positive waveform is reflective of overhydration/vascular refill—all of which are displayed in real time). In our study, all participants had a PS reduction of at least 10% from baseline. The majority (83%) of the population spent half of treatment with a PS reduction of 20%, with further reduction of 40% (consistent with previous findings) (26). Various studies have described the impact of changing circulating volume (due to HD/ultrafiltration) on myocardial blood flow and identified the development of ischemic injury during HD (17, 21, 22, 24, 37)—broadly described as occurring at the end of HD—as a time of heightened circulatory stress and volume depletion (17, 21, 22, 24). However, current findings indicate that at the microcirculatory level, the lowest PS reduction was reached after only 2 hours of HD, when approximately 50% of the target volume removal was achieved. This early signal may be a warning of pending circulatory stress and supports literature describing the microcirculatory response as preceding that of the macrovasculature (7, 8). This mid-treatment threshold may be a key timepoint that is worth further consideration or evaluation in the future. In addition, although statistical significance was not reached due to our small sample size, participants with more severe myocardial stunning (i.e., 7–12 RWMAS developed) had a lower PS reduction than those with fewer RWMAS. They also spent more time at each PS threshold, had a lower reduction in global myocardial perfusion. and had been on dialysis for a longer period of time (HD vintage).

Since there were no associations (in this study or the previous pilot study) between the development of HD-induced circulatory stress and routinely measured intradialytic monitoring parameters nor symptoms of hemodynamic instability, our study suggests that there is a benefit to incorporating microcirculatory monitoring into routine HD care. This alternative perspective may provide valuable insight into a patient’s individual response to treatment.

### 4.1 Limitations

Intradialytic imaging is extremely difficult to incorporate into research and, although our study cohort was small, the results (indirect *vs*. direct perfusion) are impactful. PPG technology does have some limitations. Since the skin is thermally regulated, it is unknown if ambient room temperature or comfort measures (warm blankets) had any impact on the PPG waveforms. In addition, changes in patient positioning plays a role in PPG outputs; for example, the reclined position increases pre load, whereas the standing position decreases pre load.

### 4.2 Conclusion

In conclusion, our study shows that the skin (an accessible microcirculatory vascular bed) is a surrogate for direct measures of organ perfusion. PPG technology is a well-accepted option that can be used for enhanced intradialytic monitoring at the microcirculatory level, providing a window of opportunity for the preemptive adapting of therapy and individualizing of treatment. This proactive approach may result in safer HD delivery, with improved clinical outcomes where current methods fail.

## Data availability statement

The raw data supporting the conclusions of this article will be made available by the authors, without undue reservation.

## Ethics statement

The studies involving human participants were reviewed and approved by Western University Health Science Research Ethics Board. The patients/participants provided their written informed consent to participate in this study.

## Author contributions

JDP, LH, and CWM contributed to conception and design of the study. JDP organized the study database. FRS, DW, and MHJ carried out the coding for raw data analysis. LH was the sole contributor to CT data acquisition and analysis. JDP conducted the statistical analysis. JDP wrote the first draft of the manuscript. LH and FRS wrote sections of the manuscript. All authors contributed to the article and approved the submitted version.
